# Obituary

**Published:** 1851-03

**Authors:** 


### ARTICLE VIII.

### OBITUARY.

Died in Chicago, February 18, of Typhoid Fever, Mr. N. C. Wells, Medical Student in Rush Medical College, from Hancock County, Illinois. Mr. Wells had earned a good reputation as a student and gentleman amongst his acquaintances in this city.

Died in Paris, December 15, 1850, Royer Collard, M. D., Professor of Hygiene, in the Faculty of Medicine.

